# Comparison of Impressions of COVID-19 Vaccinations Stratified by the Number of Vaccinations Among Japanese Healthcare Professional University Students

**DOI:** 10.7759/cureus.55861

**Published:** 2024-03-09

**Authors:** Akihiro Yokoyama, Hiromi Suzuki, Hiroaki Kataoka, Yoshiro Mori, Yuji Watanabe, Nobuyuki Miyatake

**Affiliations:** 1 Department of Hygiene, Kagawa University, Miki, JPN; 2 Department of Physical Therapy, Okayama Healthcare Professional University, Okayama, JPN; 3 Department of Pharmaceuticals, Sakaide City Hospital, Sakaide, JPN; 4 Department of Occupational Therapy, Okayama Healthcare Professional University, Okayama, JPN

**Keywords:** university students, correspondence analysis, text mining, vaccinations, covid-19

## Abstract

Objective: Coronavirus infectious disease, that emerged in 2019 (COVID-19) has been a major public health issue not only in Japan, but also worldwide, and the implementation of a proper vaccination strategy has been important. To promote vaccination, the present study compared impressions of COVID-19 vaccinations stratified by the number of vaccinations among healthcare professional university students in Okayama, Japan, and suggests better vaccination strategies.

Method: A total of 212 Japanese healthcare professional university students were enrolled in this clinical qualitative study using the text mining method. A self-reported questionnaire, including questions such as “What do you think about COVID-19 vaccinations?” was performed. We also examined the number of vaccinations, sex, history of COVID-19 infection, and daily mask use.

Results: A total of 5,935 words were obtained and “Think” (169 times) was the most frequently used followed by “Inject” (108 times), “Inoculation” (97 times), “Vaccine” (83 times), “Corona” (66 times) and “Side effects” (49 times). Characteristic words were “Safety” in non-vaccinated subjects and “Side effects” and “Necessary” in vaccinated subjects. In addition, “Safety” in non-vaccinated men and “Frightening” in non-vaccinated women were characteristic and fundamental features.

Conclusion: Impressions of COVID-19 vaccinations stratified by the number of vaccinations differed among healthcare professional university students. The provision of appropriate information on safety to non-vaccinated subjects and side effects to vaccinated subjects appears to be necessary. In addition, sex-specific information may be required for non-vaccinated subjects.

## Introduction

Coronavirus infectious disease, which emerged in 2019 (COVID-19) was initially reported in 2019 and became a major public health issue worldwide [1-3]. The first case of COVID-19 reported in Japan was in 2020, and approximately 33 million individuals have been infected with COVID-19 as of May 2023 [4,5]. COVID-19 vaccinations are recognized as one of the most effective strategies for preventing COVID-19, and approximately 78.3% of the Japanese population has been vaccinated at least once [6]. The COVID-19 classification was changed from category 2 (i.e., Avian influenza H5N1) to category 5 (i.e., influenza) in infectious law by the Ministry of Health, Labour and Welfare, Japan on May 8, 2023. However, COVID-19 infections continue to be reported [7], and the rate of vaccination in Japan is lower in younger subjects than in older subjects [8].

Previous studies examined impressions of COVID-19 vaccinations mostly using a quantitative method [9-11]. The text mining method, a qualitative method, has been employed to investigate impressions of a number of diseases, including, human papillomavirus [12,13], depression [14], atopic dermatitis [15], and many others [16]. A text mining analysis is a method of analyzing text, among other qualitative data, and is being actively examined in the field of information science [17]. In a text mining analysis, words are automatically extracted from text data and subdivided into smaller units, such as sentences and words. The structure and elements of the text are then adjusted to a form that is easier to understand. An exploratory analysis using a number of statistical methods is performed, with the aim of discovering knowledge from within the text, such as relationships and similarities between sentences and words [18,19].

Despite the usefulness of text mining, few studies have examined impressions of COVID-19 vaccinations using text this method [20-25], particularly in Japan. We previously investigated the pre-impressions of medical staff towards the first, second [23], and third [24] COVID-19 vaccinations in a designated medical institution for Class Ⅱ infectious diseases and found that the younger generation were concerned about side effects and a negative impact on pregnancy in the first COVID-19 vaccination, while the expression of concerns about side effects was reduced in the second COVID-19 vaccination [23]. We also compared impressions of COVID-19 and influenza vaccinations by analyzing social media (twitter®) using text mining [25]. However, evaluations of impressions of COVID-19 vaccinations stratified by the number of vaccinations using the text mining method were not fully discussed, particularly in the younger generation in their 20s in Japan.

Therefore, to promote proper COVID-19 vaccination strategies in the future including other vaccinations, we compared impressions of COVID-19 vaccinations stratified by the number of vaccinations among healthcare professional university students in Japan.

## Materials and methods

Materials

**Figure 1:**
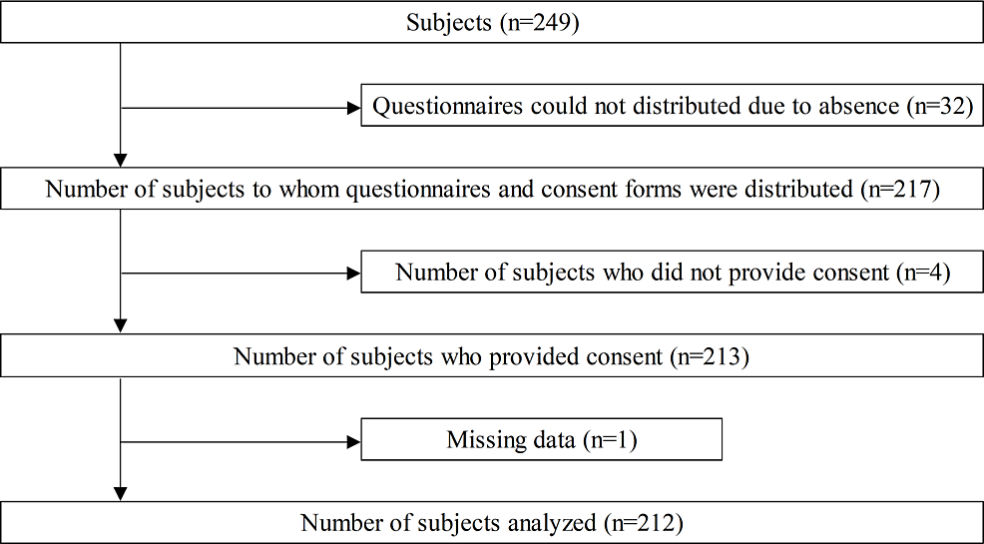
Selection process for the target population

A total of 212 students (85.1%) (116 men and 96 women, 19.7±1.1 years) who provided their written informed consent were enrolled in this cross-sectional study (Figure 1). Non-probability sampling was used and the sampling was during the late pandemic period. The Institutional Review Board for Experimental Research on Human Subjects at Okayama Healthcare Professional University issued approval 0071.

Questionnaire

We evaluated impressions of COVID-19 using a self-reported questionnaire, which included questions such as “What do you think about COVID-19 vaccinations?” between May 23 and June 29, 2023. We obtained information on the number of COVID-19 vaccinations ("How many doses of COVID-19 vaccine have you received?"), sex (men or women), age (years), school year (grade), a history of COVID-19 infection (yes or no), and daily mask use (yes or no). This questionnaire was performed using Google forms® [26] with a QR code. Questions were answered from students’ own smartphones and the questionnaire took between 10 minutes and 15 minutes to complete.

Statistical analysis

Data are expressed as the number of subjects and percentages for sex, age, school year, the number of COVID-19 vaccinations, a history of COVID-19 infection, and daily mask use. Fisher’s exact test was used to examine differences by sex and number of vaccinations, by a history of COVID-19 infection and the number of vaccinations, and by the mask-wearing status and number of vaccinations, with p<0.05 indicating a significant difference. Data were analyzed using JMP Pro 17 (SAS Institute Inc., Cary, USA).

Self-reported texts regarding impressions of COVID-19 vaccinations were analyzed using text mining software (KH Coder 3.0, Koichi Higuchi, Tokyo, Japan) [27,28]. Words were automatically extracted from the sentences collected by Google form® using KH Coder to create a list of the frequency of their occurrence. KH Coder was performed with default settings to eliminate the bias of the analyst’s opinion as much as possible, and default settings were also used for the morphological analysis dictionary. Regarding coding rules, the words “corona” and “corona vaccine” were divided and analyzed. Regarding the way these words were used in the text, we performed a morphological analysis and confirmed the relationship between the words before and after corona and vaccine using the keyword in context method [29]. Using a correspondence analysis, which visualizes the relationship between words in scatter plots, we analyzed data by sex, the number of COVID-19 vaccinations, a history of COVID-19 infection and number of COVID-19 vaccinations, and by daily mask use and the number of COVID-19 vaccinations. Extracted words were translated into English using DeepL® translation [30].

## Results

Table 1 shows the clinical profiles of enrolled subjects (Table 1). The number of COVID-19 vaccinations was as follows: 0, 16 subjects (7.5%); 1-2 times, 68 subjects (32.1%); 3 times, 82 subjects (38.7%); 4 times or more, 46 subjects (21.7%). We compared the number of subjects stratified by sub-categories (sex and the number of COVID-19 vaccinations, a history of COVID-19 infection and the number of COVID-19 vaccinations, and daily mask use and the number of COVID-19 vaccinations) (Table 2). Significant differences were observed between the number of subjects stratified by a history of COVID-19 infection and the number of COVID-19 vaccinations.

**Table 1:** Clinical profiles of enrolled subjects (n=212)

	Number of subjects	%
Sex		
Male	116	54.7
Female	96	45.3
Age		
18	37	17.5
19	57	26.9
20	70	33.0
21	43	20.3
22	4	1.9
26	1	0.5
Year		
1st year	41	19.3
2nd year	67	31.6
3rd year	74	34.9
4th year	30	14.2
Number of vaccinations		
0 times	16	7.5
1-2 times	68	32.1
3 times	82	38.7
4 times or more	46	21.7
Infection		
Previously infected	95	44.8
Never infected	117	55.2
Daily mask use		
Yes	169	79.7
No	43	20.3

**Table 2:** Comparison by vaccination frequency Fisher's direct probability test (*p*<0.05) Bold values indicate *p*<0.05

Number of vaccinations	0 times	1-2 times	3 times	4 times or more	*p*-value
Sex					
Male	7	40	46	23	0.6300
Female	9	28	36	23	
Infection					
Previously infected	10	40	32	13	0.0033
Never infected	6	28	50	33	
Daily mask use					
Yes	15	47	68	39	0.0604
No	1	21	14	7	

We then performed a text mining analysis (Table 3). The total number of words obtained was 5,935 words, with “Think” (169 times) being the most frequent, followed by “Inject” (108 times), “Inoculation” (97 times), “Vaccine” (83 times), “Corona” (66 times) and “Side effects” (49 times).

**Table 3:** Frequently occurring words (total of 5935 times)

	Word	Number of times	%
1	Think	169	2.85
2	Inject	108	1.82
3	Inoculation	97	1.63
4	Vaccine	83	1.40
5	Corona	66	1.11
6	Side effects	49	0.83
7	Person	41	0.69
8	Infection	38	0.64
9	Necessary	29	0.49
10	Frightening	26	0.44
11	Virus	22	0.37
11	Good	22	0.37
13	Reaction	20	0.34
14	Effect	18	0.30
14	Consider	18	0.30

**Figure 2:**
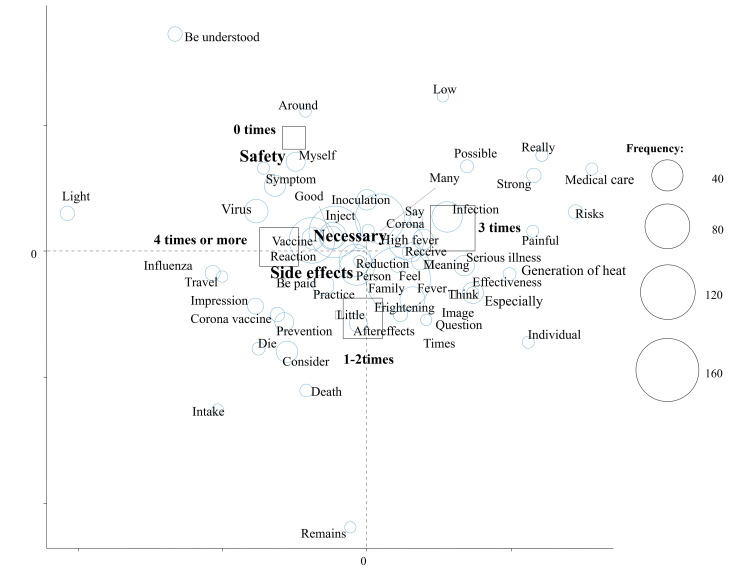
Correspondence analysis of responses by the number of vaccinations

**Figure 3:**
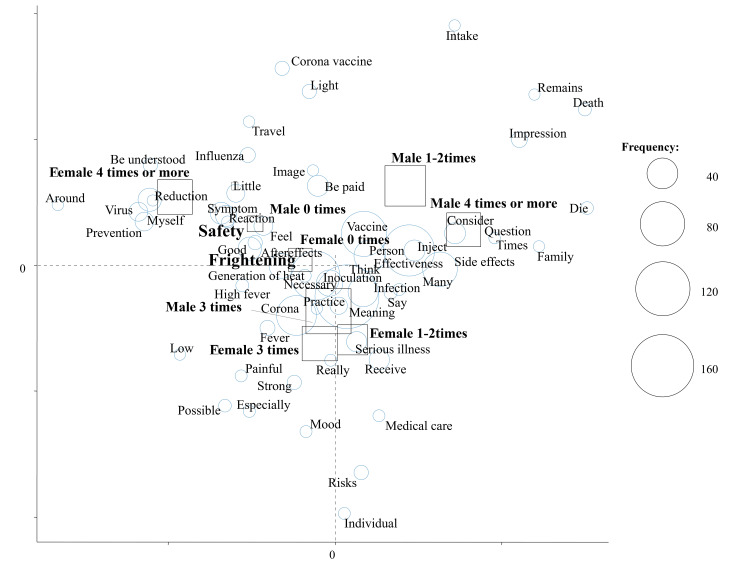
Correspondence analysis by sex and the number of vaccinations

**Figure 4:**
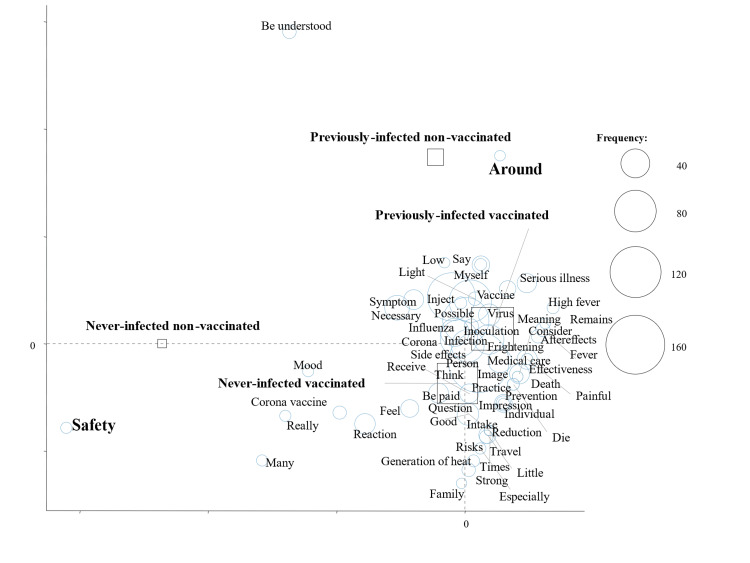
Correspondence analysis by the infection status and vaccination status

**Figure 5:**
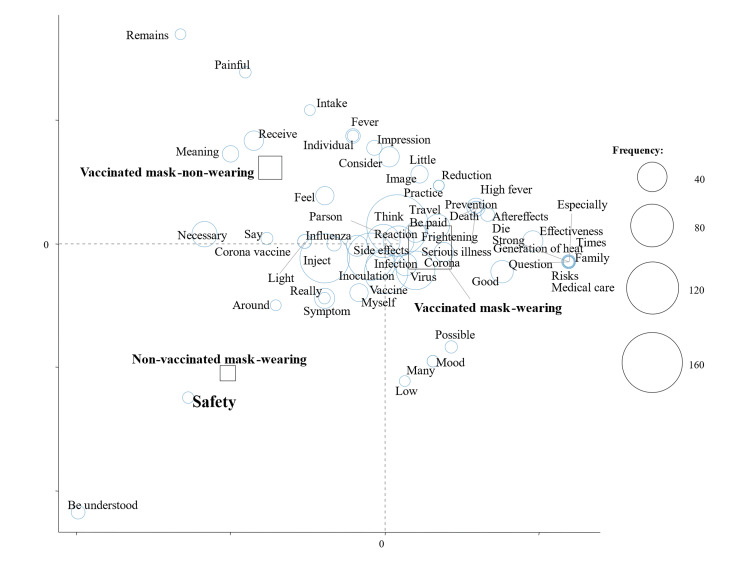
Correspondence analysis by daily mask use and the vaccination status

Figure 2 shows the results of a correspondence analysis stratified by the number of COVID-19 vaccinations; characteristic words were “Safety” in non-vaccinated subjects and “Side effects” and “Necessary” in vaccinated subjects (Figure 2). We also performed a correspondence analysis stratified by subcategories (Figures 3-5). “Safety” in non-vaccinated men and “Frightening” in non-vaccinated women were characteristic and fundamental features stratified by sex and the number of vaccinations (Figure 3). Characteristic words were “Safety” in never-infected non-vaccinated subjects and “Around” in previously infected non-vaccinated subjects stratified by a history of COVID-19 infection and the number of vaccinations (Figure 4). We then evaluated characteristic words using a correspondence analysis stratified by the mask-wearing status and number of vaccinations (Figure 5). “Safety” was characteristic in non-vaccinated mask-wearing subjects (Figure 5).

## Discussion

In the present study, we initially compared impressions of COVID-19 vaccinations stratified by the number of vaccinations among Japanese healthcare professional university students. We found that impressions of COVID-19 vaccinations stratified by the number of vaccinations markedly differed, with characteristic words of “Safety” in subjects and “Side effects” and “Necessary” in vaccinated subjects using a text mining analysis.

Previous studies examined impressions of various diseases using a text mining analysis [12-16]. Okuhara et al. evaluated changes in impressions of HPV vaccinations in newspaper text using a cluster analysis and correspondence analysis and found a marked change in keywords over time [13]. Falissard et al. [15] investigated the effects of atopic dermatitis on daily life in 1231 patients using a co-occurrence network in a cross-sectional study. Relationships were observed between negative elements in daily life and keywords such as “Itch,” “Pain,” and “Embarrassment” [15]. Atkinson-Clement et al. found that the severity of tics and “Comorbidities” affected the daily social life of patients with Tourette syndrome using a correspondence analysis [16]. These studies performed a text mining analysis, to identify specific words and relationships among these words from a different viewpoint to a conventional qualitative analysis. In the present study, we initially compared impressions of COVID-19 vaccinations by healthcare university students in Okayama prefecture, Japan using text mining, particularly a correspondence analysis.

Previous studies investigated impressions of COVID-19 vaccinations using a qualitative method [9-11]. Muscillo et al. evaluated impressions of COVID-19 vaccinations in 395 Italian high school students using a selective questionnaire in a cross-sectional study and men with vaccinations were found to have become more pessimistic [9]. In Japan, Kajiwara et al. investigated the reasons for hesitations to get vaccinated in 1020 women aged 15-49 years using a selective questionnaire [10]. Okamoto et al. also examined impressions of COVID-19 vaccinations in 2160 university students and 2289 teachers and staff using a selective questionnaire via e-mail [11]. These studies employed a selective questionnaire and there were a number of limitations, such as selective bias and accuracy.

On the other hand, some studies, including those by our group [23-25], examined impressions of COVID-19 vaccinations using a text mining analysis [20-22]. Lim et al. used a self-reported questionnaire through Google Form® in 284 adults, and “Anxious,” “Afraid,” and “Scared” were commonly detected words [20]. Qorib et al. analyzed tweets from Twitter® and detected changes in impressions of COVID-19 vaccinations over time [21].

Collectively, these findings suggest that the text mining method is more useful for analyzing impressions of COVID-19. The clinical significance of the present study is that a questionnaire on impressions of COVID-19 vaccination was administered to students at a healthcare professional university using our previously established research methodology and presented in a text mining analysis by the number of vaccinations. In the present study, impressions of the COVID-19 vaccine differed with the number of COVID-19 vaccinations. Therefore, it appears to be important to provide a detailed explanation of safety to non-vaccinated subjects and side effects to vaccinated subjects as well as to inform them of the necessity for vaccinations. In addition, an explanation of safety may be more useful, for non-vaccinated men, while strategies to cope with fear may be more useful for non-vaccinated women. A safety explanation may be important even for never-infected non-vaccinated subjects and non-vaccinated mask-wearing subjects. “Around” was a characteristic word in previously infected non-vaccinated subjects, and, thus, an accurate explanation of vaccinations may be necessary not only for the subjects themselves but also for those around them.

There are a number of limitations that need to be addressed. Enrolled subjects (non-probability sampling) may have been more health-conscious than average university students. In addition, 85.1% of students completed the analysis in this survey. The characteristics of non-respondents may have differed from those of respondents, and it may affect the results. Therefore, it may not be possible to apply the results of the present study to all university students. Moreover, this was a cross-sectional, not longitudinal study. In addition, analyses of the questionnaire survey took approximately one month to complete. As Qorib et al. reported, opinions and impressions rapidly change over time [21,22]. Therefore, it is possible that the impressions of subjects changed during that time. Nevertheless, the present results provide useful information for promoting future vaccination strategies including vaccines other than that for COVID-19 vaccination.

## Conclusions

Marked differences were observed in impressions of COVID-19 vaccinations stratified by the number of COVID-19 vaccinations among Japanese healthcare university students. Safety warrants further consideration among non-vaccinated subjects and side effects among vaccinated subjects. In addition, sex-specific information may be required for non-vaccinated subjects.
